# Skin Protective Nutraceuticals: The Current Evidence in Brief

**DOI:** 10.3390/healthcare6020040

**Published:** 2018-05-04

**Authors:** Oroma Nwanodi

**Affiliations:** Obstetrics and Gynecology Locum Tenens, Salinas, CA 93902, USA; o.nwanodi@juno.com; Tel.: +1-314-304-2946

**Keywords:** atopic dermatitis, green tea, human skin, keratinocyte, moisturizer, nutraceuticals, photoprotection, polyphenols, probiotics, vitamin E

## Abstract

Nutraceuticals are important for healthy skin maintenance. Probiotics, phenolics, and vitamins are just a few of the nutraceuticals meant to potentially prevent and assist medical management of dermatologic conditions. Among these, probiotics, vitamin E, and green tea catechins may offer the broadest array of skin protective mechanisms with probiotics having the greatest clinical range. Probiotics’ amelioration of atopic dermatitis and opportunistic infections of skin burns has been targeted in recent research efforts. This includes the improvement of Scoring Atopic Dermatitis index scores, *p* = 0.02, with intact *Lactobacillus rhamnosus* Goldin and Gorbach (LGG) in comparison to heat inactivated LGG or placebo. *Lactobacillus reuteri* used prior to or concurrently with *Staphylococcus aureus* infection can increase epidermal keratinocyte survival, *p* < 0.01. Phenolics may not have been extensively studied for atopic dermatitis or skin burns. However, phenolics do have a role in photoprotection. The phenolic rutin increases ultraviolet B radiation filter reactive oxygen species scavenging at 75%, *p* < 0.002, and peak wavelength absorption, *p* < 0.001. While oral and topical probiotics have untapped potential for atopic dermatitis amelioration and skin infection prevention, phenolics will be increasingly used for photoprotection. With optimized bioavailability, dosage, and formulation, nutraceuticals will become crucial for healthy skin maintenance.

## 1. Introduction

Nonmelanoma skin cancers (NMSC), which are comprised primarily of basal cell carcinomas (BCC) and squamous cell carcinomas (SCC), are one of the most common human cancers with an 18-fold to 20-fold higher incidence than melanoma [1,2]. Therefore, photocarginogenesis prevention is important. Global NMSC incidence varies. BCC is most common in Australia and the United States of America (USA) at more than 1000 and 450 per 100,000 person-years, respectively [2]. BCC is the least documented in Africa at less than 1 per 100,000 person-years [3]. Meanwhile, SCC occur at one-third the rate of BCC, which amounts to 22.65 per 100,000 person-years in England [2,3]. Annually, at least 1.3 million NMSC occur in the USA, affecting one in five Americans and costing more than $650 million USD [4]. The skin, which is a multifunctioning organ, is the innate photocarginogenesis prevention organ. The skin controls body temperature and water content, which prevents dryness. The skin protects the covered body from infection, physical damage, and ultraviolet radiation (UVR) [5]. Damage to the skin occurs via several modalities including heat, moisture, UVR, and direct mechanical trauma. UVR from a 200 nm to 400 nm spectrum has dose-dependent photo-carcinogenic and photo-damaging effects including immunosuppression, UVR-dependent acute inflammation with dermal leukocytic infiltrate, and erythema (sunburn) [6]. Acute inflammation induces cytosolic phospholipase A2, which increases free arachidonic acid, the substrate for eicosanoid production by cyclooxygenases and lipoxygenases, and leads to prostaglandin E2 (PGE2) and 12-lipoxygenase-derived 12-hydroxyeicosatetraenoicacid (HETE), which are the two primary actors in a sunburn [6].

Ultraviolet A radiation (UVA), which has a 320 nm to 400 nm spectrum range, is the deepest penetrating UVR at 1000 μm into the epidermis and dermis and comprises 90% to 95% of UVR [1]. UVA dysregulates melanogenesis via oxidative stress induction and antioxidant defense impairment while ultraviolet B radiation (UVB) and ultraviolet C radiation (UVC) directly damages DNA [1,7]. UVA activates melanogenesis, increases tyrosinase concentration and activity, and increases reactive oxygen species (ROS) and 8-hydroxy-2′–deoxyguanosine (8-OHdG) while reducing glutathione S-transferase (GST) [7]. Within one hour after exposure, UVA is associated with a decreased nuclear factor E2-related factor 2 (Nrf2) nuclear translocation and transcriptional activity. UVA exposure is associated with glutamate cysteine ligase catalytic subunit (GCLC), GST, and nicotinamide adenine dinucleotide phosphate quinone oxidoreductase 1 (NQO1) downregulation. UVA suppression of extracellular signal-related kinases (ERK), Jun nuclear kinase (JNK), and p38 mitogen-activated protein kinases (MAPK) concurrently decreases Nrf2 nuclear translocation [7].

Keratinocytes are the major cells of the epidermis, which is the outermost skin layer [8]. The most differentiated keratinocytes are found in the stratum corneum outer horny layer and least differentiated keratinocytes are found in the stratum basale closest to the dermis [8]. Being primarily connective tissue, the dermis contains collagen, elastin, fibroblasts, and immune cells [8]. The underlying hypodermis provides vascular and neural networks to the dermis and epidermis [8]. An intact stratum corneum—outer horny skin layer—is elastic and hydrated. Cracking, pruritus, and scaling are signs of a dehydrated, inelastic, damaged outer horny skin layer, which is a skin barrier disorder.

Increased outer horny skin layer thickness is associated with atopic dermatitis (eczema; AD) [5]. About 14% of children under four years of age have AD [9]. Globally, up to 20% of persons may experience AD in their lifetime [10]. Annually in the USA, AD costs about $1 billion USD [10]. Externally applied moisturizers improve dry skin’s barrier function and reduce inflammation, which forms part of AD treatment [5,9]. Skin barrier disorders such as AD also have immunologic components [5]. Extrinsic or allergic AD accounts for up to 80% of AD and involves IgA, IgE, and regulatory T-cells [9]. The hygiene hypothesis posits that the cleanliness-based lack of antigen exposure decreases self-reactive T-cell suppression, leading to increased autoimmunity and immunologic system-based disorders and includes atopic dermatitis [11,12]. The revised hygiene hypothesis is more specific, linking early childhood impaired immune system development and the hygiene hypothesis with an altered intestinal microbiome. This, in turn, increases allergic and autoimmune disease susceptibility [11,12]. Consistent with the hygiene hypothesis, decreased microbial antigen exposure as an AD risk factor has been strengthened by the association of filaggrin mutation and increased *Staphylococcus aureus* in 42% of AD patients [10]. Consistent with the revised hygiene hypothesis’ importance of the intestinal microbiome composition, breastfeeding has been associated with a 54% reduction in AD for 13,557 term infants [12,13]. In contrast to formula-fed children, breast-fed children have a *Bifidiobacterium* (Acintobacteria)-based intestinal microbiome [12,13].

Although Hippocrates publicized foods’ medicinal value, nutraceutical is a twentieth century term [14]. Based on the European Nutraceutical Association definition, nutraceuticals are naturally occurring, non-toxic, bioactive compounds from animal, microbial, and plant sources that have disease prevention, health promotion, or medicinal properties [14,15]. While nutraceuticals can offer an alternative to synthetic pharmaceuticals, in addition to being components of nutritive, functional foods, nutraceuticals may be used as pharmaceutical formulations [14,15]. Particularly when consumed as foods, nutraceuticals may have lower bioavailability and poorer tissue distribution than pharmaceuticals [14]. Nutraceuticals are most effective when hepatic phase II detoxification metabolism and the gastrointestinal microbiota function appropriately [14,16]. Numerous skin protective nutraceutical categories exist. The focus henceforth will be on probiotics, polyphenols, and vitamins. The literature for aesthetic skin nutraceuticals contains several other categories including carotenoids, organosulfurs, polysaccharides, polyunsaturated fatty acids, quinones, and retinoids. Of these, carotenoids and polyunsaturated fatty acids will be succinctly mentioned [4,17].

## 2. Probiotics

Long-term skin commensal microbiome stability dominated by the Actinobacteria *Propionibacterium* and *Corynebacterium,* Firmicutes *Staphylococcus,* Proteobacteria and Bacteroidetes is achieved in childhood [10]. Skin commensals directly protect against pathogenic microbes and modulate type 1 T-helper (Th1) and type 2 T-helper (Th2) mediated immune responses [10]. Skin commensals’ composition is varied in AD [10]. Stable *S. aureus* colonization with less than 10^6^ cfu/cm^2^ of skin occurs in 20% of people and intermittent carriage occurs in 60% of people [8,18]. Normally, *S. aureus* at greater than or equal to 10^6^ cfu/cm^2^ of skin is considered an infection [8]. However, in heat damaged (burned) skin as well as in cracked, dehydrated AD skin, *S. aureus* causes dose dependently severe opportunistic infections from 10^5^ cfu/mL or greater [18]. Therefore, 80% of people are at risk of opportunistic *S. aureus* infections with any skin barrier disruption, which occurs with burns, AD, and acne vulgaris [18]. In addition, altered skin commensal bacterial microflora are associated with AD and acne vulgaris [18].

Probiotics are live, active, microbial cultures that are generally regarded as safe (GRAS) when consumed in quantities that achieve health benefits to the host [8,9]. Heat-inactivated or killed bacteria are not probiotics. Probiotics have not shown any benefit that improves preterm infants’ gastrointestinal barrier function and overall health [19]. Probiotics neutralize pathogenic microbes by enhancing hosts’ mucin secretions that trap pathogens by acid and bacteriocin secretion as well as by increasing ceramide production without harming the host [8,20,21]. Probiotics also protect from pathogenic microbes through competitive exclusion at cell receptor binding sites and competitive nutrient consumption [8,18]. Probiotics can modify and regulate host innate immune responses for additional protection against pathogenic bacteria [8]. Probiotic lactobacilli including *Lactobacillus plantarum* have shown nonspecific immune system boosting and regulation [5,20]. Lactobacilli protect phagocytes from pathogen-directed apoptosis [20]. Therefore, probiotics perform the same anti-pathogenic and anti-inflammatory immunologic functions as the skin commensal microbiome. As such, it is biologically plausible that, when the skin commensal microbiome composition is changed in AD and heat damaged skin, probiotics can function in lieu of the normal skin commensal microbiome.

Historically, with oral formulations, probiotics had to survive gastric transport, adhere to, and colonize the gastrointestinal tract [21]. These criteria are redundant for topically applied probiotics. A topical combination of *Lactobacillus paracasei* and *Bifidobacterium longum reuter* reportedly decreased leg dryness and facial roughness in 33 adult females over an eight-week trial [8]. Subsequently, a 66-female double-blind, placebo-controlled, randomized trial of *B. longum reuter* lysate (disrupted bacteria) topically applied to reactive skin twice daily for two months achieved increased skin barrier resistance measured by reduced trans-epidermal water loss (TEWL) and reduced skin sensitivity and dryness in response to thermal and chemical physical changes [22].

Based on a meta-analysis of 19 trials totaling 4076 intervention and 3700 control events, respectively, maternal probiotic consumption in late pregnancy and, during lactation, could reduce AD incidence in children younger than five years old, Risk Ratio 0.78, 95% Confidence Interval (CI) 0.68 to 0.90, I^2^ = 61%, Absolute Risk Reduction 44 cases per 1000 children; 95% CI 20 to 64 [23]. A systematic review of 12 case-control studies on probiotics’ effect in pediatric AD patients in Europe and Australia found probiotics to be beneficial [9]. Nine of the included studies showed an association between probiotic use and reduced infections, improved immune response, and reduced gastrointestinal inflammation [9]. *Lactobacillus rhamnosus* Goldin and Gorbach (LGG), when not heat inactivated, significantly improves the Scoring Atopic Dermatitis index (SCORAD) scores in comparison to heat inactivated LGG or the placebo, *p* = 0.02 [9]. LGG also improves SCORAD scores in IgE-sensitized AD patients, *p* = 0.036 [9]. A placebo-controlled trial of 1 × 10^9^ cfu, oral *Lactobacillus fermentum* VRI-033 PCC, twice daily for 8-weeks achieved SCORAD improvements with sustained T-helper 1 IFN-γ responses, *p* = 0.046 for 2-months post-treatment, which were directly proportional to the SCORAD score improvement [9]. However, *L. fermentum* VRI-003 use was not associated with reduced topical corticosteroid use [9].

A *Lactobacillus salivarius* synbiotic (probiotic with prebiotics to increase probiotic delivery) was trialed against prebiotic only in sixty 2- to 14-year-old children with moderate to severe AD [10]. At eight weeks and 10 weeks, *L. salivarius* synbiotic recipients had significantly lower SCORAD scores (27.4 ± 12.7) than prebiotic recipients (36.3 ± 14.9; *p* = 0.022) [10,24]. In adults, oral *L. salivarius* LS01 has been shown to preserve Th1 cytokine production and Th1:Th2 ratios when compared to maltodextrin controls after four months of treatment [25]. *L. salivarius’* reduction of AD severity via normalization of T-cell function is consistent with the hygiene hypothesis [11].

A *Bifidobacterium longum infantis* and *B. breve* combination in fermented infant formula achieved greater IgA responses to the poliovirus vaccine challenge than standard formula [25]. A *L. salivarius* and *B. breve* BR03 combination led to improved SCORAD indices in adult AD patients [25]. *B. breve* Bb99 synbiotic with three additional probiotics given to 459 women during the last two to four weeks of pregnancy and given to their newborns for six months after delivery reduced AD in comparison to 463 controls (Odds Ratio (OR) = 0.74; 95% CI, 0.55–0.98, *p* = 0.04) [24]. Similarly, an *L. rhamnosus* LCS-742 and *B. longum infantis* M63 synbiotic administered to 39 term newborns for six months reduced AD in comparison with 45 formula-fed control newborns (OR = 0.11; 95% CI, 0.01–0.94; *p* < 0.05) [24]. Overall, mixed strain synbiotics appear to be more effective for AD treatment than single strain synbiotics, *p* = 0.03, based on a SCORAD-weighted mean difference improvement of 7.32 for 80 treated participants from three studies [24]. Consistent with this, a trial of *B. breve* M-16V synbiotic versus placebo control in 90 infants less than seven months of age did not find differences in cytokine production or circulating regulator T-cell proportions [26]. However, a mother and infant double-blinded, placebo-controlled trial of LGG, *L. rhamnosus* LC705, *B. breve* Bb99, and *Propionibacterium freudenreichii* spp. Shermanii JS, with 1223 subjects found that in cesarean delivered infants, IgE-associated allergic diseases at five years of age were significantly reduced (24.3% versus 40.5%, OR = 0.47; 95% CI, 0.23–0.96%, *p* = 0.035) [27]. It has been postulated that only IgE-associated AD will respond to probiotics [12].

Lysates offer cost-savings over the use of selected single components. Lysates can be included in prepared foods. A randomized controlled trial (RCT) with 41 dry, dark-skinned 25- to 60-year-old patients tested orally consumed 2.1% *L. plantarum* K8 (KCTC 10887BP) lysates. The 2.1% *L. plantarum* K8 lysates increased forearm skin hydration by the fourth week (*p* = 0.03), but not after eight weeks of use (*p* = 0.068) [5]. However, facial skin hydration was increased at four weeks (*p* = 0.00) and eight weeks (*p* = 0.007) of use [5]. Orally consumed 2.1% *L. plantarum* K8 lysates were associated with significantly decreased horny layer thickness of the forearm and face at four weeks and eight weeks with *p* = 0.002 and *p* = 0.000 for the face and *p* = 0.007 and *p* = 0.000 for the forearm [5]. Orally consumed 2.1% *L. plantarum* K8 lysates use was associated with statistically significant decreased TEWL of the forearm and face at eight weeks with *p* = 0.002 and *p* = 0.008, respectively [5]. Therefore, 2.1% *L. plantarum* K8 lysates may be regarded as orally consumed skin moisturizers that reduce the need for topical corticosteroids and emollients for AD treatment [5].

*Pseudomonas aeruginosa* is known for opportunistic infections in burn patients through the use of a toll-like receptor-4 (TLR-4) associated lipopolysaccharide receptor complex to induce an inflammatory and adaptive immune response [20]. *L. plantarum,* 10^5^ cfu/mL injected into infected areas on days three, four, five, seven, and nine post infection inhibited *P. aeruginosa* PA100 colonization of an in vivo burned-murine model, which was measured by the lack of viable *P. aeruginosa* PA 100 cells, *p* < 0.001 [20]. *L. plantarum* was tried against silver sulphadiazine in 80 patients with delayed, infected second-degree and third-degree burns, or early, non-infected third-degree burns [28]. Within treatment groups of 12 to 15 patients, outcomes lacked statistical power. Nonetheless, when compared to silver sulphadiazine, *L. plantarum* achieved a 2.72% lower bacterial load in second-degree burns and 1.07% less infection in early third-degree burns, but had 16.90% more infection in delayed, infected third-degree burns [28]. These results were consistent with rabbit models [29]. 

*Lactobacillus reuteri* ATCC 55730 at 10^5^ to 10^8^ cfu/mL and *L. rhamnosus* AC413 coinfections increase normal primary human epidermal keratinocytes (NHEK) survival in the presence of concurrent *S. aureus* infection from 8.8% in the absence of probiotics to 53.1% (*p* = 0.0012) and 42.7% (*p* < 0.0001), respectively [18]. *L. reuteri* ATCC 55730 lysate also protects NHEK from *S. aureus*, *p* = 0.01. Heat-killed *L. reuteri* ATCC 55730 is ineffective against *S. aureus* infection [18]. *L. reuteri* ATCC 55730 exposure after *S. aureus* infection is ineffective. *L. reuteri* ATCC 55730 introduction before or concurrently with *S. aureus* allows *L. reuteri* ATCC 55730 to competitively exclude *S. aureus* from NHEK binding sites, *p* = 0.026 and *p* = 0.0078, respectively [18]. Compared to live *L. reuteri* ATCC 55730, *L. reuteri* ATCC 55730 lysates are less effective staphylococcal adhesion inhibitors, *p* = 0.0002 versus *p* = 0.032 [18]. *L. salivarius* UCC118 cannot be substituted for *L. reuteri* ATCC 55730 since *L. salivarius* UCC118 adheres less effectively to NHEK than does *L. reuteri* ATCC 55730, 5.6 ± 0.1 log cfu versus 6.7 ± 0.1 log cfu respectively, *p* = 0.005 [18].

In vitro studies show that LGG lysates and spent culture fluid can protect human keratinocytes from subsequent *S. aureus* exposure with *p* = 0.006 and *p* = 0.01 after a 24-h incubation [8]. LGG and LGG lysates were also effective with exposure limited to two hours before or 12 h after *S. aureus* infection, *p* = 0.005 and *p* = 0.01, respectively [8]. Live LGG and LGG lysate physically displace *S. aureus* from receptors [8]. Live LGG, LGG lysate, and spent culture competitively exclude *S. aureus* from keratinocytes’ receptors [8]. Live LGG and LGG lysate inhibited *S. aureus* growth. However, spent culture did not inhibit *S. aureus* growth [8].

## 3. Polyphenols: Flavonoids, Phenolic acids, Stilbenes, and Proanthocyanidins

Pigmented polyphenols absorb UVR primarily in the UVB spectrum with some UVA and UVC absorption, which facilitates sunscreen function [1]. Globally, *Camellia sinensis* derived tea is 20% green tea and is the second most common drink [30,31]. Topical green tea extract is known to reduce UVR-induced p53 expression [1]. Epigallocatechin-3-gallate (EGCG) has been associated with impaired in vitro nucleotide biosynthesis repair via dihydrofolate reductase inhibition [30]. In murine models, topical resveratrol, silymarin, oral resveratrol, silymarin, and grape seed proanthocyanidins inhibit UVB-induced photodamage. This anti-inflammatory action mediates polyphenols’ anti-photo-carcinogenesis [1,4].

### 3.1. Flavonoids

Anti-inflammatory, antioxidant, polyphenolic green tea catechins (GTC) inhibit cyclooxygenase-2 and lipoxygenases [6]. Conjugates and metabolites of orally consumed GTC are incorporated into the skin and have been found in skin biopsy specimens [6]. In murine trials, oral GTC and EGCG protected against UVR-induced photodamage and photocarcinogenesis [1,6]. EGCG is the primary catechin and the most bioactive constituent of green tea [31,32]. In open, uncontrolled human trials, topical GTC preceding UVR exposure reduced photodamage and oral GTC reduced photodamage by increasing the skin erythema (sunburn) threshold [6]. In controlled human trials, EGCG (<1 mg/cm^2^) preceding UVR exposure also reduced photodamage [1]. A 34-day, 39-person, controlled trial of topical 4% green tea extract (OM24) compared to GTC preceding UVB exposure found sunburn reduced by 38.9% by OM24, *p* = 0.02 [33].

Oral GTC metabolites are incorporated into the skin [6]. Oral GTC were studied in a three-month, double-blind, RCT, with 50 female and male 18- to 65-year-old patients, Fitzpatrick skin phototypes I to II representing northern Europeans and Scandinavians [6]. Based on the placebo control, GTC dosed at 1080 mg/d with vitamin C 100 mg taken daily did not significantly reduce skin erythema, leukocyte infiltration, or eicosanoid response to a UVR inflammatory challenge [6]. The GTC was the equivalent of five cups of green tea daily: comprised of 12.6 mg catechin, 1.8 mg catechin gallate, 75 mg epicatechin, 295.8 mg epigallocatechin, 156 mg epicatechin gallate, 435.6 mg EGCG, 1.8 mg gallic acid, 74.4 mg gallocatechin, and 28 mg gallocatechin gallate. The median interquartile range sunburn threshold (minimal erythema dose) was 20 mJ/cm^2^ to 28 mJ/cm^2^ in both intervention and placebo groups [6]. Post-UVR neutrophil and CD3^+^ T-lymphocyte counts were similar in each group, *p* = 0.85 and *p* = 0.62, respectively [6]. The lack of significant outcomes may be due to under dosing GTC. Men and women who drink more than 10 cups of green tea (equivalent to 2.5 g of green tea extract) daily have delayed cancer onset by 3.2 years and 7.3 years, respectively [30].

Topical green tea EGCG effects IL-12-dependent DNA repair [30]. However, topical EGCG is subject to photolysis (76.9 ± 4.6%) [32]. α-Lipoic acid more effectively reduces photolysis of combined EGCG in comparison with vitamin C (12.6 ± 1.6%, *p* < 0.05 versus 20.4 ± 2.7%) [32]. α-Lipoic acid also most effectively preserves EGCG’s antioxidant activity (−1.4%) [32]. Interestingly, vitamin E increases photolysis of combined EGCG to 84.5 ± 3.4%, *p* < 0.05 [32].

### 3.2. Phenolic Acids

Dietary phenolic acids are primarily UVA absorbing antioxidants with indirect regulatory effect on the nuclear factor E2-related factor 2-antioxidant responsive element (Nrf2-ARE) pathway [7]. In primary human epidermal melanocytes (HEMn) and B16F10 melanoma cells, Nrf2 inhibits melanogenesis and tyrosinase protein expression. Caffeic acid and ferulic acid are partial UVA protectors. Quercetin and rutin are strong UVA protectors. Avobenzone is an efficient UVA filter lacking antioxidant activity [7]. Normally, B16F10 melanoma cells increase melanin production, tyrosinase activity, and tyrosinase protein expression in response to UVA. B16F10 melanoma cells pretreated with caffeic acid, ferulic acid, quercetin, rutin, and avobenzone display inhibited melanin production and tyrosinase activity. Quercetin is most effective, IC30 7.8 ± 1.4 μM against melanin production and 10.1 ± 3.1 μM against tyrosinase activity [7]. Rutin, caffeic acid, and avobenzone have equivalent, less effective activity than quercetin, *p* < 0.05, *p* < 0.01, and *p* < 0.001, respectively. Ferulic acid is least active with IC30 > 30 against melanin production and tyrosinase activity [7]. Nonetheless, quercetin, caffeic acid, and avobenzone may reverse UVA-mediated Nrf2 nuclear translocation downregulation, Nrf2-ARE downregulation, and GCLC, GST, and NQO1 reduction [7]. Although less effective than quercetin, when rutin is added to UVB filters, ethylhexyl methoxycinnamate, ethylhexyl dimethyl PABA, and octocrylene organic UV filters, ROS scavenging is increased by 75% (*p* < 0.002) and peak wavelength absorption is increased (*p* < 0.001) [34]. Rutin increases the critical wavelength of ethylhexyl dimethyl PABA (*p* ≤ 0.05) [34]. UVA photo-protection of ethylhexyl methoxycinnamate and ethylhexyl dimethyl PABA is increased with the addition of rutin (*p* ≤ 0.05) [34]. Of note, caffeic acid, rutin, and quercetin are found in South African *Aspalathus linearis* (rooibos) tea. Caffeic acid and ferulic acid are constituents of *Polypodium leucotomas*, a tropical fern [35]. *P. leucotomas* extract is commercially available in several products [35]. In a small 9-person study, oral *P. leucotomas* extract significantly reduced tumorigenesis and immunosuppression associated cyclobutane pyrimidine dimers formed by UVB action on DNA, *p* < 0.001 [35]. This finding is consistent with the literature on *P. leucotomas* [36].

### 3.3. Stilbenes and Proanthocyanidins 

Photoprotection and anti-photocarcinogenesis murine trials of resveratrol abound [37]. In vivo murine studies of oral grape seed proanthocyanidins show a dose-dependent anti-photo-carcinogenesis effect [38]. In vivo human trials of oral resveratrol and grape seed proanthocyanidins have been hampered by poor bioavailability [33,37]. Cosmeceutical patents have been awarded based on in vitro human cell line studies [39]. In response, topical delivery mechanisms including hydrogel patches, nano suspensions, co-loaded liposomes, and co-loaded glycerosomes have been developed [37,40]. Nonetheless, topical formulations face bioavailability issues. The active constituents must penetrate and diffuse through the stratum corneum into the stratum basale [16].

Silymarin is up to 80% silibinin is GRAS [4]. In addition to anti-inflammatory photoprotection, silymarin has antioxidant and immune modulating mechanisms of action. In vivo murine models show topical silibinin and oral grape seed proanthocyanidins have antioxidant properties by inhibiting MAPK ERK and p38 proteins and NF- κβ activation [4,38]. Topical silibinin inhibits UVR-induced immune suppression by inhibiting leukocyte infiltration especially in MHC + CD 1 1b+ cell types [4]. In vitro A431 human epidermoid cancer cell line studies show that silibinin induces autophagy by limiting UVB-induced apoptosis [41].

## 4. Vitamins C and E

Vitamins C and E are normal skin components [42]. UVR-induced erythema is unchanged, but skin and plasma vitamin C content rises with 500 mg daily oral vitamin C supplementation [42]. Up to 10% topical vitamin C neither irritates nor sensitizes human skin [42]. Vitamin E is currently understood to be comprised of α-, β-, δ-, and γ-tocopherols and α-, β-, δ-, and γ-tocotrienols [43,44]. Importantly, tocopherols and tocotrienols have distinct bioactive compartments of distribution [43,44]. Although vitamin E inactivation of ROS from UVR exposure is consistent with dose-dependent α-tocopherol depletion by UVB with lipophilic penetration in the brain and liver and even intramembrane positioning close to membrane surfaces, tocotrienols are more powerful antioxidants than α-tocopherol [42,43]. Tocotrienols have anti-inflammatory properties that tocopherols lack [43]. Tocotrienols induce antioxidant enzymes, antigen specific, and antigen nonspecific immune responses [43]. Tocotrienols are cyclooxygenase and 5-lipoxygenase-catalyzed eicosanoids inhibitors, which suppress nuclear factor-κβ, a signal transducer, and activation of transcription (STAT) pro-inflammatory signaling [43]. Therein lies additional biologic basis for vitamin E limiting photo- damage.

Vitamin E comprised 30% of mixed tocopherols and 70% of mixed tocotrienols and a water-soluble phenolic-flavonoid-rich antioxidant complex are minor components of *Elaies guineensis* (West African palm fruit), which was transported globally by the Portuguese in the 15th Century and the Dutch in the 19th Century [45]. *Elaeis oleifera* (South American palm fruit) has more oleic and linoleic acid and less palmitic and saturated acids than *E. guineensis* [45]. *E. guineensis* tocotrienols are unusual since rice bran oil is the only other tocotrienol containing vegetable oil [45]. Palm oil has a higher mixed tocopherol and mixed tocotrienol content (693.5 mg/kg) than sunflower oil (547.5 mg/kg) or olive oil (175.6 mg/kg), but less than rice bran oil (1198 mg/kg), soybean oil (902.2 mg/kg), corn oil (781.4 mg/kg), or wheat germ oil (702.7 mg/kg) [45]. However, except for rice bran and wheat germ oil, these other oils lack tocotrienols [45]. Tocotrienols have multiple anti-cancer mechanisms of action: malignant cell proliferation and 3-hydroxy-3-methyl-glutaryl-coenzyme A (HMG-CoA) reductase suppression, malignant cell apoptosis induction, cell cycle arrest, immunity enhancement, angiogenesis, and tumor cell migration inhibition [43]. Consistent with this, palm oil vitamin E, which is a water-soluble phenolic-flavonoid-rich antioxidant complex, and carotenoids have demonstrated anti-glioblastoma, anti-neuroblastoma, and an anti-breast, gastric, pancreatic cancer effect in murine models [43,45]. Nano emulsified Malaysian palm oil tocotrienol fraction has been patented with the antioxidant, soy isoflavone, Genistein for slow-release liquid and cream skin photo-protection applications [46]. In vitro, there is a 2.7-h drug release lag time [46]. Cell viability following UVB-exposure reached 80%, *p* < 0.03, when compared to control cells [46].

## 5. Other Supplements—Carotenoids, Coenzyme Q10, Polyunsaturated Fatty Acids

Based on 20 healthy adults, an oral combination of 25 mg total carotenoids and 335 mg (500 International Units) *RRR*-α-tocopherol daily for 12-weeks reduced dorsal skin erythema (*p* < 0.01) by week nine in comparison to 25 mg total carotenoids daily [47]. Combinations of β-carotene, lycopene (a lipophilic carotenoid), and lutein can provide equivalent photoprotection to higher doses of β-carotene used alone [48]. This is important for smokers and those exposed to asbestos who must limit β-carotene intake [48]. A commercially available combination of lycopene 2.5 mg, β-carotene, and *Lactobacillus johnsonii* 5.10 × 10^8^ cfu was the intervention in a 12-week randomized, double-blinded, placebo-controlled trial with 60 patients who had polymorphic light eruptions [49]. The combination of lycopene 2.5 mg, β-carotene, and *L. johnsonii* 5.10 × 10^8^ cfu was able to reduce the effect of a single exposure to longwave UV radiation (UVA1), *p* < 0.001, but that of multiple exposures [49]. UVA1-inducible gene mRNA expression is a surrogate marker for UVA exposure [48,49]. Intracellular adhesion molecule-1 mRNA levels are associated with polymorphic light eruptions following UVA1 exposure, *p* < 0.001, and *p* = 0.022 in the placebo group [49]. Lycopene 16 mg from tomato paste 55 g in olive oil or olive oil only were consumed daily by 20 women with FitzPatrick skin phototypes I or II in a 12-week, RCT [50]. Tomato paste increased the mean erythemal dose by 30 units (*p* = 0.03), increased procollagen deposition (*p* = 0.05), reduced UVR-induced matrix metalloproteinase-1 (*p* = 0.04), and reduced mitochondrial DNA 3895-base pair deletion after 3× minimal erythema dose UV exposure (*p* = 0.01) [50]. Olive oil carrier was used for increased lycopene absorption [50]. A 5 mg lycopene with tomato phytonutrient complex including tocopherols and phytosterols soft gel capsule (TNC) or 10 mg free lutein were stabilized with 10% carnosic acid soft gel capsule (LCC) dosed as 2-TNC twice daily or 1-LCC twice daily, which were trialed against a soybean oil soft gel capsule placebo [48]. Based on 65 participants in a 28-week long, double-blind, randomized, crossover trial, when TNC or LCC were received from day 1, there is similar UVA, UVB, and longwave UVA1 protection [48]. The doses of TNC and LCC used correspond to 130 g of chopped kale and 242 g of tomato juice [48].

The antioxidant, coenzyme Q10 is essential for mitochondrial energy metabolism [51]. In contrast to the literature of in vitro studies, a 33-person double-blind, placebo-controlled study of 50 mg and 150 mg of oral coenzyme Q10 daily for 12-weeks did not find a significant increase in the minimal erythema dose (MED). At 12 weeks, the placebo MED was 0.62 J/cm^2^ ± 0.04, 50 mg coenzyme Q10 MED was 0.72 J/cm^2^ ± 0.06 and 150 mg coenzyme Q10 MED was 0.70 J/cm^2^ ± 0.06, *p* = 0.49 [51].

A 3-month, 42-person, double-blind randomized trial of omega-3 polyunsaturated fatty acids 4 g daily, eicosapentaenoic acid (EPA), or monounsaturated oleic acid found that EPA was the most promising for photo-protection [52]. EPA increased the UV-induced erythemal threshold by 13 mJ/cm^2^, *p* < 0.01, and reduced p53 UV-induced skin expression by 50%, *p* < 0.01 [52]. This was a follow-up to a successful trial of 10 g fish oil daily for three months or six months in which the MED was more than doubled [53].

## 6. Discussion

The preceding sections and the synopsis of selected references in Table 1 show a broad array of skin protective nutraceuticals. For different pathologic processes, a given nutraceutical can be effective through different mechanisms of action, as indicated in Table 2. Multiple mechanisms of action allow probiotics to be effective for immunologic-based AD, prevent skin dehydration and infections, and promote wound healing [5,8,9,12,18,27]. Given an expanding body of evidence, over time, recommendations supporting maternal oral probiotic consumption during pregnancy and lactation have gained strength [12,13,23,27]. A person’s microbiota is partially determined by delivery route [27]. Oral maternal combination probiotics are a means to give persons delivered by cesarean a similar microbiome as those delivered vaginally. *L. salivarius* symbiotic should be considered for AD treatment given reduced AD severity measured by SCORAD, *p* = 0.022 [10]. Probiotic formulation affects efficacy. *B. breve* may need additional trials as a combination probiotic in fermented foods before significant effects on AD was seen [25]. The combination probiotic LGG, *L. rhamnosus* LC705, *B. breve* Bb99, and *Propionibacterium freudenreichii* spp. Shermanii JS, reduced IgE-associated allergic diseases in cesarean-delivered infants (*p* = 0.035), but *B. breve* as a single administered probiotic did not affect cytokine production or circulating regulator T-cell proportion [26,27]. Successful probiotic use in IgE-associated AD stimulates toll-like receptors by modulating cytokines and inducing IgA [27]. Future probiotic trials for AD need subgroup analysis for IgE-associated AD and for birth by cesarean delivery [12,27].

Orally consumed 2.1% *L. plantarum* K8 lysates are skin moisturizers, which increase facial skin hydration through eight weeks of use (*p* = 0.007), decrease the horny layer thickness of the forearm and face through eight weeks of use (*p* = 0.000 for the face and forearm), and decrease TEWL of the forearm and face at eight weeks (*p* = 0.002 and *p* = 0.008, respectively) [5]. Therefore, orally consumed 2.1% *L. plantarum* K8 lysates should reduce the need for topical corticosteroids and emollients for AD treatment [5]. Decreased horny layer thickness is important since this denotes a shift towards normal skin [5,8]. Given some directly equivalent outcomes to topical silver sulphadiazine, topical *L. plantarum* is a candidate for further larger scale in vivo study as a topical burn treatment [28,29]. Ideally, probiotic exposure should precede or be concurrent with pathogen exposure. Probiotic exposure after pathogen exposure may not prevent opportunistic infections [8,18].

Live or lysate probiotic preparations are biologically plausibly efficacious where heat-killed preparations are ineffective [8,18]. Live cultures can competitively deplete the pathogens’ nutrients. Live cultures are more proficient with competitive receptor binding and receptor binding displacers than are heat-killed or heat-inactivated substances [8,18]. As with heat-inactivated LGG for AD amelioration, heat-killed *L. reuteri* ATCC 55730 for *S. aureus* opportunistic infection ensures the effectiveness of the non-denatured probiotic [9,18]. Therefore, it has been recommended that probiotics or lysates, but not heat-inactivated substances, become components of barrier creams and soaps used by persons with propensity for skin disruption to reduce pathogenic microbe skin colonization and ensuing opportunistic infections [11].

Oral and topical polyphenols hold the photoprotection and anti-photocarginogenesis promise. Additional human trials on GTC and EGCG are needed. Formulations with improved GTC and EGCG bioavailability such as combinations with α-lipoic acid may allow anti-inflammatory photo protective effectiveness at doses equivalent to less than 10 cups of green tea daily [6,30,32]. Historic bioactivity work on vitamin E focused on α-tocopherol by discounting the role of tocotrienols. However, there is a growing body of literature that indicates that tocotrienols have powerful anti-inflammatory photoprotection activity and lipophilic penetration into the brain and liver distinct from tocopherols [43,44]. Therefore, the need to study full spectrum palm oil vitamin E and water-soluble phenolic-flavonoid-rich antioxidant complex and carotenoids further is supported by the literature [47]. Similarly, quercetin, resveratrol, silymarin, and grape seed proanthocyanidins should progress from murine to human trials. Larger scale human trials of β-carotene, lycopene, and lutein with tomato phytonutrients, coenzyme Q10, and EPA should be considered.

## Figures and Tables

**Table 1:** Selected reference synopsis.

**Skin, Photodamage, Photoprotection, Anti-Photocarginogenesis, and Atopic Dermatitis**		
**Ref.**	**Type**	**Primary Results**
[1]	Review	Describes the effects of different solar ultraviolet radiation spectra on the skin, which leads to photocarginogenesis.
[5]	Experimental	Describes skin function and the physiologic basis of atopic dermatitis. Suggests probiotics positively affect lipoteichoic acid a cell wall component.
[8]	Experimental	One-chapter description of skin structure and barrier function, wound formation and immunologic response, and probiotics’ roles in human health.
**Probiotics**		
**Ref.**	**Type**	**Primary Results**
[11]	Review	Hygiene hypothesis originated from the inverse correlation between hay fever and the number of older siblings in British children. Extended to autoimmune disorders: infections against the basis of epidemiological data, animal models, and human intervention trials. Infections are protective against many immune-related disorders. Lack of infections ↑ T helper type 1, ↓ T helper type 2 cells, favors strong immune responses from weak antigens, and is consistent with increasing incidence of atopic dermatitis, asthma, and allergic rhinitis. Probiotics and microbiota are weak antigens and non-antigenic ligands that alter immuno-regulatory mechanisms. Probiotics and microbiota modulate immune responses.
[12]	Review	Reviews atopic dermatitis epidemiology and pathophysiology, hygiene hypothesis, intestinal microbiome, probiotics, prebiotics, and synbiotics.
[30]	Review	Epidemiology and cost of nonmelanoma skin cancer in the United States of America. Reviews ultraviolet radiation’s photo-damaging effects and topical sunscreens’ limitations.
[5]	Experimental	Describes relationship betweeen probiotics, gastrointestinal microbiota, and atopic dermatitis incidence. Randomized controlled trial of orally consumed *Lactobacillus plantarum* K8 (KCTC 10887BP) 2.1% lysates, with 6-weeks old SKH-1 hairless mice and 41-participants aged 25 to 60 years old. In vivo, hydration ↑ in the intervention group on the face after four and eight weeks (*p* = 0.000, *p* = 0.007, respectively). Horny layer thickness ↓ at four weeks and eight weeks for the face (*p* = 0.002, *p* = 0.000) and the forearm (*p* = 0.007 and *p* = 0.000). Transepidermal water loss ↓ on the face and forearm at eight weeks (*p* = 0.008, *p* = 0.002, respectively). Orally consumed *L. plantarum K8* lysates improved skin barrier function.
[8]	Experimental	Doctoral thesis. In vitro effect of probiotics on *Staphylococcus aureus* infected wounds. Live *Lactobacillus rhamnosus* GG ↑ infected human keratinocytes’ viability from 25 to 57% at 24-h of incubation. *L. rhamnosus* GG lysates and spent culture fluid also protected keratinocytes (*p* = 0.006, *p* = 0.01).
[9]	Review	Twelve case-control studies from Australia and Europe. Brief decription of atopic dermatitis and Scoring Atopic Dermatitis (SCORAD) evaluation system. Nine studies found probiotics to be beneficial in atopic dermatitis by providing infection prevention, improved immunologic response with a bifidobacteria altered gut microbiome, and reduced inflammation. Outcomes: Oral *Lactobacillus rhamnosus* GG use reduces SCORAD (*p* = 0.02). Four weeks of oral *L. rhamnosus* GG reduced intestinal inflammation in children with atopic dermatitis and allergy to cow’s milk. In a population in which 71% had high IgE food antigen responses, oral *L. fermentum* VRI-003 reduced moderate and severe atopic dermatitis (SCORAD improvement *p* = 0.03).
[10]	Review	Probiotics given to young infants may prevent atopic dermatitis development. Prebiotic-controlled trial of *L. salivarius* in 60 children, 2-years-old to 14-years-old, found improved atopic dermatitis at 8-weeks and 10-weeks (SCORAD improvement *p* = 0.022). Placebo-controlled trial of *L. plantarum* CJLP133 in 118 children, 1-years-old to 13-years-old, found improved atopic dermatitis at 14-weeks (SCORAD improvement *p* = 0.044).
[24]	Meta-analysis	Six treatment studies (369 children, 0-years-old to 14-years-old) and two prevention studies (1320 children, 0-months-old to 6-months-old) of synbiotic use in atopic dermatitis. The treatment studies were heterogenic (I^2^ = 77.1%, *p* = 0.001). Mixed strain synbiotics were beneficial (*p* = 0.03). Synbiotics were beneficial for children at least 1-year-old (*p* = 0.048). Synbiotics were not shown to prevent atopic dermatitis (*p* = 0.26).
[25]	Review	Intestinal microbiota composition associated with atopic dermatitis: Neonates who develop atopy have different microbiota to neonates who do not develop atopy. Probiotics affect T-regulatory and T-helper cells, augment IgA responses, and evolve adaptive T-cell immunity.
**Probiotics**		
**Ref.**	**Type**	**Primary Results**
[27]	Experimental	Double-blind, placebo-controlled study of combined strain probiotic (2 lactobacilli, 1 bifidobacteria, and 1 propionibacteria) with 1223 mothers with infants from the last month of pregnancy through six-month-old infants. Five-year visit data outcomes analysis: probiotic receiving cesarean-delivered children had less IgE-associated allergic diseases (24.3% versus 40.5%; odds ratio 0.47; 95% confidence interval, 0.23 to 0.96%, *p* = 0.035).
[28]	Experimental	Eighty burn patients treated with *L. plantarum* or silver sulphadiazine. *L. plantarum* competes with pathogenic bacteria, alters the wound microenviroment, and promotes tissue repair. Demonstrated the equivalence of *L. plantarum* to silver sulphadiazine.
**Polyphenols**		
**Ref.**	**Type**	**Primary Results**
[16]	Review	Chemical classification, physicochemical properties, transportation through the skin, metabolism, and physiological mechanisms of action (anti-inflammation, estrogen-like, anti-infective, and anti-aging).
**Flavonoids**		
**Ref.**	**Type**	**Primary Results**
[1]	Review	Mechanisms of selected polyphenols’ photo-protective and anti-photo-carcinogenic effects: green tea polyphenols, grape seed proanthocyanidins, resveratrol, silymarin, and genistein. Primarily anti-inflammatory and antioxidant. Plasma bioavilability limited by conjugation to glucuronide, sulphate, and methyl groups. As water additives, green tea polyphenols protect against skin tumorigenesis in murine models. Topically photo-protective in human models, it reduces erythema, inflammation, and tissue infiltration. Topical and oral epigallocatechin-3-gallate are photo-protective.
[6]	Experimental	50-participant, double-blind, randomized, placebo-controlled trial of 1350 mg green tea catechins and 50 mg vitamin C twice daily for three months, clinicaltrials.gov registration number NCT01032031. Minimal erythemal dose was not significantly different across groups. Similar skin neutrophil, CD3^+^ T-lymphocytes, cyclooxygenase, and lipoxygenase metabolites prostaglandin E2, and 12-hydroxyeicosatetraenocacid across groups.
[30]	Review	Classification of polyphenol constistuents of green tea. Green tea polyphenol use, limitations, biologic plausibility, mechanisms of action, and potential photoprotection and anti-photo–carinogenesis role.
[31]	Review	Mechanism of action based on medicinal application of green tea’s constituents for photoprotection, dermatologic, and infectious disease treatment.
[32]	Experimental	In vitro photo-degradation studies of 1% epigallocatechin-3-gallate emulsions with and without equimolar co-oxidants α-lipoic acid, butylated hydroxytoulene, vitamin C, and vitamin E. Vitamins C and α-lipoic acid reduced photodegration of epigallocatechin-3-gallate from 76.9 ± 4.6% to 20.4 ± 2.7% and 12.6 ± 1.6%, respectively while vitamin E increased photodegration to 84.5 ± 3.4%. An antioxidant activity decrease was at the lowest value with α-lipoic acid, 1.4%, making α-lipoic acid the best of the four trialed co-oxidants to stabilize 1% epigallocatechin-3-gallate emulsions.
**Flavonoids**		
**Ref.**	**Type**	**Primary Results**
[33]	Review	Seven human subjects and eight murine studies encompass polyphenols from *Calluna vulgaris*, cocoa, green or white tea, grape seeds, honeybush, *Lepidium meyenii* (maca), and Romanian propolis. Efficacy, the mechanism of action, and adverse effects of various polyphenol formulations need further study.
**Phenolic Acids**		
**Ref.**	**Type**	**Primary Results**
[7]	Experimental	In vitro, primary human epidermal melanocyte and B16F10 melanoma cell trial of caffeic acid, ferulic acid, quercetin, rutin, and avobenzone. All five phenolics showed anti-melanogenic effects in reversal of ultraviolet-A radiation-mediated oxidative damage and downregulation of Nuclear factor E2-related factor 2 activity in B16F10 cells. This section discusses oxidative stress mechanisms.
[34]	Experimental	In vitro synergism of rutin with ultraviolet filters (ethylhexyl methoxycinnamate and ethylhexyl dimethyl para-aminobenzoic acid) from reactive oxygen species scavenging (also synergistic with octocrylene), prevents sunscreen photolysis and increases ultraviolet-A critical wavelengths with photoprotective gain (*p* ≤ 0.05).
[35]	Review	Caffeic acid and ferulic acid containing *Polyodium leucotomos* is photoprotective and anti-photocarcinogenic via anti-inflammatory, antioxidant, antitumorigenic, and immunoregulatory mechanisms. Oral *P. leucotomos* is a potential systemic photoprotective and anti-photocarcinogenic agent.
[36]	Systematic review	Four studies suggest that *P. leucotomos* is photoprotective. Three studies found that *P. leucotomos* delayed or prevented polymorphous light eruptions or solar urticaria. One study found a trend toward reduced ultraviolet radiation induced DNA mutations following exposure to two or three times the minimal erythemal dose (*p* = 0.06 and *p* = 0.07, respectively).
**Stilbenes**		
**Ref.**	**Type**	**Primary Results**
[1]	Review	Topical and oral silymarin and silibinin are anti-photocarcinogenic in murine models.
[41]	Experimental	In vitro study showed that silibinin-induced autophagy photoprotects human epidermoid carcinoma A431 cells from ultraviolet-B radiation, which induced apoptosis via the insulin growth factor-1 Receptor-Protein kinase B (IGF-1R-Akt) pathway activation. Rapamycin is an autophagy inducer enchanced silibinin’s effects.
**Proanthocyanidins**		
**Ref.**	**Type**	**Primary Results**
[1]	Review	Grape seeds as dietary additives protect from skin tumorigenesis in murine models. Polymeric with limited gastrointestinal absorption.
**Proanthocyanidins**		
**Ref.**	**Type**	**Primary Results**
[38]	Review	*Vitis vinifera* (Grape) seeds proanthocyanidins are the main red wine polyphenol. These are more effective antioxidants and reactive oxygen species scavengers than vitamins C and E. For anti-photocarcinogenesis use, mitogen-activated protein kinases and nuclear factor-κB signaling pathways and immunosuppression through alterations in immunoregulatory cytokines. In vitro and in vivo murine studies suggest that dietary grape seed proanthocyanidins should undergo human trials.
[40]	Experimental	In vitro study of resveratrol and gallic acid co-loaded in phospholipid vesicles dispersed in water-propylene glycol or water-glycerol liposomes. Gallic acid accumulated in the skin, keratinocytes, and fibrobasts were protected from oxidative damage and antimicrobial activity was shown.
**Vitamins C and E**		
**Ref.**	**Type**	**Primary Results**
[32]	Experimental	In vitro photodegradation studies of 1% epigallocatechin-3-gallate emulsions with and without equimolar co-oxidants α-lipoic acid, butylated hydroxytoulene, vitamin C, and vitamin E. Vitamins C and α-lipoic acid reduced photodegration of pigallocatechin-3-gallate from 76.9 ± 4.6% to 20.4 ± 2.7% and 12.6 ± 1.6%, respectively. Antioxidant activity decreased the least with α-lipoic acid, 1.4%.
**Vitamins C and E**		
**Ref.**	**Type**	**Primary Results**
[42]	Review	Describes ultraviolet radiation’s effect on the skin and the skin’s antioxidant defenses: *β*-carotene, Coenzyme Q10, glutathione, green tea, retinoids, Vitamin C, and Vitamin E.
[43]	Review	Describes the differences between tocotrienols and tocopherols by focusing on tocotrienols action against chronic diseases in contrast to α-tocopherol’s. Reactive nitrogen species scavenging, cyclooxygenase- and 5-lipoxygenase-catalyzed eicosanoids inhibition, and proinflammatory signalling suppression: nuclear factor-κB and signal transducer and activation of transcription (STAT) action. Tocotrienols’ pharmacology, metabolism, toxicology, and biosafety are discussed.
[44]	Review	Focus on cardioprotection. Reviews sources of toctrienols, bioavailability, and antioxidant effects.
[45]	Review	Palm fruit chemistry: tocotrienol, carotenoid-rich, saturated and monounsaturated fatty acids, antioxidant and anti-cancer effects including those of a water-soluble phenolic-flavonoid-rich complex.
**Carotenoids**		
**Ref.**	**Type**	**Primary Results**
[47]	Experimental	12-week, dual intervention, randomized, self-controlled study with 20 participants, 20-years old to 57-years old, Fitzpatrick skin phototypes I or II. *Dunaliella salina* derived 20% carotenoid mixture (primarily β-carotene) in soybean oil with 3% to 5% algal sterols and hydrocarbons dosed at 25 mg carotenoids daily and 25 mg carotenoids with 335 mg *RRR*-α-tocopherol daily formed the interventions.
[47]	Experimental (continued)	The carotenoid and *RRR*-α-tocopherol intervention achieved greater eythema suppression than carotenoid only intervention. Both groups had yellowing of the skin primarily of the palms and face.
[49]	Experimental	Randomized, placebo-controlled, double-blinded study with 60 adult polymorphic light eruption patients, Fitzpatrick skin phototypes I, II, or III, non-obese, and <125 g/day fermented food intake. Lycopene 2.5 mg, β-carotene 4.7 mg, and *Lactobacillus johnsonii* 5 × 10^8^ cfu, nutritional supplement reduced polymorphic light eruption score after a single ultraviolet-A1 exposure (*p* < 0.001) but not after two exposures. The intervention increased intracellular adhesion molecule 1 mRNA expression (*p* = 0.022).
[50]	Experimental	Randomized, olive-oil controlled study with 20 women, 21-years-old to 47-years-old, Fitzpatrick skin phototypes I or II. Lycopene 16 mg in 55 g tomato paste in olive oil was consumed daily for 12 weeks. The intervention increased the mean erythemal D30 (*p* = 0.03), reduced matrix metalloproteinase-1 (*p* = 0.04), and reduced mitochondrial DNA 3895-basepair deletion (*p* = 0.01).
**Coenzyme Q10**		
**Ref.**	**Type**	**Primary Results**
[51]	Experimental	Dual intervention, randomized, placebo-controlled study with 33 women, 45- to 60-years-old, Fitzpatrick skin phototypes II or II. The low-dose intervention was coenzyme Q10 50 mg/day and the high-dose intervention was coenzyme Q10 150 mg/day. Both low-dose and high-dose coenzyme Q10 showed skin viscoelasticity and hydration retention while the placebo did not (*p* = 0.03 and *p* = 0.06, respectively). High-dose coenzyme Q10 was protective against periorbital and upper radial lip line wrinkles (*p* < 0.05) and nasolabial fold and corner of the mouth wrinkles (*p* < 0.01). Low-dose coenzyme Q10 was protective against periorbital line wrinkles (*p* < 0.05).
**Polyunsaturated Fatty Acids**		
**Ref.**	**Type**	**Primary Results**
[52]	Experimental	Dual intervention, double-blind, randomized study, 42-subjects, 21- to 65-years-old, Fitzpatrick skin phototypes II or III. The 3-month long oral interventions were the ω-3-fatty acid eicosapentaenoic acid 4 g/day or oleic acid 4 g/day. Eicosapentaenoic acid increased the erythemal threshold (*p* < 0.01) and reduced ultraviolet radiation induced p53 exression (*p* < 0.01).
[53]	Review	The anti-inflammatory mechanism of action of *n*-3-polyunsaturated fatty acids reduced prostaglandin and cytokine production in response to ultraviolet radiation.

CD3+, cluster of differentiation three activating T-cell co-receptor; IgE, immunoglobulin E; IGF-1R-Akt, insulin growth factor-1 Receptor-Protein kinase B; Ref., references; SCORAD, scoring atopic dermatitis evaluation system; STAT, signal transducer and activation of transcription.

**Table 2:** Skin protective nutraceuticals’ mechanisms of action.

Skin Damage Form: Mechanism/Effect	Probiotics	Flavonoids Quercetin	Phenolic Acids Caffeic, Ferulic, & Gallic Acids, Rutin	Stilbenes Resveratrol Silibinin (Silymarin)	Vitamin E Tocotrienols	Carotenoids Tomato Derived	Coenzyme Q10	Eicosa-Pentaenoic Acid (EPA)	Green Tea Epigallocatechin-3-gallate
**Ultraviolet Radiation (UVR)**					**Ultraviolet Radiation (UVR)**				
Photodamage: Impaired extracellular matrix	-	-	-	Protects collagen from UVR-induced degradation [37].	↑ Collagen synthesis [47].	↓ UVR-induced fibrillin-1 reduction [50].	↑ dermal and epidermal basement membrane components Maintains viscoelasticity [51].	-	↓ UVR-induced collagenase [1].
Photodamage: Minimal erythemal dose (MED)	-	-	-	↑ MED [33].	-	Unclear [36,42].	-	↑ MED [52].	Conflicting ↑ MED [1,6,32,33].
Photodamage: UVR-induced inflammation	↓ Immune responses	Anti-inflammatory [14,16].	Anti-inflammatory [14]. ↓ UVR-induced macrophage & neutrophil infiltration [35]. ↓ UVR-induced cyclooxygenase, cytokine production [16,35]. ↓ pro-inflammatory mediator expression—tumor necrosis factor-α, nitrite oxide synthase (NOS) [35]. Preserve functional Langerhans cells [35].	Anti-inflammatory [4,14,16]. ↓ leukocyte infiltration, edema, prostaglandin metabolites, and cyclooxygenase-2, [4,37]. ↓ pro-inflammatory mediator expression–NOS [4].	Anti-inflammatory [43,44,47].	↑ intercellular adhesion molecule-1 mRNA expression [49].	-	↓ UVR-induced prostaglandins & cytokines [53].	Anti-inflammatory [1,14,16,32]. ↓ UVR-induced Langerhans and antigen presenting cell depletion [30,33]. ↓ mast cell histamine release [30]. ↓ UVR-induced cytokines, prostaglandins, & leukocytes [1,6,16,30,32].
Photodamage: Altered immune- regulation	-	-	Prevent trans-urocanic acid photoisomerization & photodecomposition [35].	↓ UVR-induced immune suppression [4].	Anti-inflammatory [43,44]. ↓ pro-inflammatory mediator expression—tumor necrosis factor, nitric oxide, cyclo-oxygenase-2, 5-lipoxygenase-catalyzed eicosanoids [43,44]. Immuno-stimulatory [43].	-	-	-	Dose-dependent ↓ UVR-induced immune-suppression [30]. Possible dose and/or gender based ↓ pro-inflammatory mediator expression—nitric oxide, hydrogen peroxide, cyclo-oxygenase-2 [6].
Photodamage: Reactive oxygen species (ROS) generation	-	Antioxidative [14].	↓ ROS production [35]. Free radical scavengers [14,34,36,40]. ↓ glutathione oxidation [35]. UVR photon acceptors [35]. ↓ UVR-induced melanogenesis and tyrosinase activity [7]. Prevent UVR-induced downregulation of nuclear factor E2-related factor 2 (Nrf) and Nrf-antioxidant response element [7].	Antioxidative [4,14,37]. ↓ UVR-induced intracellular peroxide and nitric oxide [4].	Antioxidative [32,44,45]. ↓ NOS and lipid peroxidation [43,44]. Scavenges ROS and nitrogen-based free radicals [43,46].	Antioxidative [42]. ROS scavenging [47].	↓ UVR-induced ROS production [42].	ROS scavenging [53].	Antioxidative [1,14,30,32]. ROS scavenging [1,30]. ↓ NOS and peroxide [30]. ↓ UVR-induced antioxidant enzyme depletion [30]. ↓ UVR-induced lipid peroxidase and protein oxidation [30]. Synergistic with α-lipoic acid [32].
**Ultraviolet Radiation (UVR)**					**Ultraviolet Radiation (UVR)**				
Photocarcinogenesis: DNA mutation induction → immune suppression	-	Anticancer [14].	Anticancer [14]. Activate p53 [35]. ↓ DNA oxidative base changes [7].Prevent cyclobutan pyrimidine dimer formation [35]. Remove UVR-induced photoproducts [35]. ↓ pro-inflammatory transcription factors—nuclear factor-κβ, activation protein-1 (AP-1) [35].	Anticancer [1,4,14,37]. ↓ pro-inflammatory transcription factors—nuclear factor-κβ, mitogen-activated protein kinases, AP-1 [4,37].	↓ DNA damage [44]. ↓ pro-inflammatory transcription factors—nuclear factor-κβ, signal transducer and activation of transcription [43,44]	↓ Mitochondrial DNA mutations [50].	↓ UVR-induced DNA damage [42].	EPA ↓ UVR-induced p53 activity, DNA single-strand breaks, and DNA oxidative base changes [52].	Anticancer [1,14,30,32]. ↑ DNA repair enzyme activity [1,30]. ↓ UVR- induced DNA oxidative base changes [30].↓ nuclear factor-κβ, AP-1 [6,30,31].
Photocarcinogenesis: Cell cycle and cellular integrity impairment	-	-	↓ cytoskeletal disarray & MMP [35]. Maintain cell viability [35].	↓ MMP [37]. Induces p53-mediated apoptosis [37].	↑cell cycle inhibitory protein, caspase-dependent, and independent apoptosis, ↓ cyclin dependent kinase [43].	↓ UVR-induced MMP [48,50].	↓ UVR-induced MMP [42].	-	↓ UVR-induced MMP [1,31,32,33] ↓ UVR-induced signaling protein phosphorylation [30].
**Immunologic disease: Atopic dermatitis (AD)**					**Immunologic Disease: Atopic Dermatitis**				
Immunologic changes	Oral probiotics directly affect Peyer’s patch M-cells and intestinal macrophages and dendritic cells [21]. Modulate T-regulatory & T-helper cell 1&2, cytokine, and IgA responses [9,12,25,27]. Stimulate adaptive T-cell maturation [25]. Favor a non-atopic state via microbiota shifts [5,12,25,27]. ↓ IgE-associated allergic diseases—extrinsic AD [9,12,27].	-	-	-	-	-	-	-	Anti-inflammatory [31].
Dehydrated skin: ↑ transepidermal water loss	Improves barrier function [5]. ↓ transepidermal water loss [5].	-	-	-	-	-	-	-	-
Dehydrated skin: ↑ stratum corneum thickness	↓ stratum corneum thickness [5].				-	-	-	-	-
Dehydrated skin: ↑ opportunistic infections	↓ opportunistic infections [10]. ↓ bacterial load [28]. Competitively exclude pathogens from keratinocytes [8].	Anti-microbial [16].	Antimicrobial [40].	-	Antibacterial [45].	-	-	-	Antimicrobial [31].
Severity Scoring of Atopic Dermatitis Index	↓ by *Lactobacillus rhamnosus* GG or *L. fermentum* VRI-003 [9]. ↓ by mixed strain use in ≤1-year-olds [24].	-	-	-	-	-	-	-	-
**Trauma—Thermal burns**					**Trauma—Thermal burns**				
Impaired wound healing	Improved wound healing [20] ↑ keratinocyte proliferation & migration [8]. ↑ reepithelization [8]. ↓ apoptosis [20]. Promote granulation tissue wound bed [28]. ↓ scarring by ↓ total collagen & ↓Type 1 collagen mRNA [29].	-	-	-	-	-	-	-	-
↑ opportunistic infections	↓ opportunistic infections [20,29]. ↑ phagocytosis, ↓ pathogen virulence factors [20]. Competitively exclude pathogens from keratinocytes [8,18]. ↓ bacterial load [28].	Anti-microbial [16].	Antimicrobial [40].	-	Antibacterial [45].	-	-	-	-

AD, atopic dermatitis; AP-1, nuclear activation protein-1; IgA, immunoglobulin A; IgE, immunoglobulin E; MED, minimal erythemal dose; MMP, matrix metalloproteinases; NOS, nitric oxide synthase; NRF, nuclear factor E2-related factor 2; ROS, reactive oxygen species; UVR, ultraviolet radiation.

## Supplementary Materials

The following are available online at http://www.mdpi.com/2227-9032/6/2/40/s1, Abbreviations List.
