# Supplementary material for: Skin Protective Nutraceuticals: The Current Evidence in Brief
Source: Healthcare (Basel). 2018 May 4;6(2):40. doi: 10.3390/healthcare6020040 (PMC6023352; doi:10.3390/healthcare6020040)
Supplement: Supplementary file 1 [file healthcare-06-00040-s001.pdf]

Abbreviations

A431 cells: Human epidermoid carcinoma cell line

AD: Atopic Dermatitis

BCC: Basal cell carcinomas

cfu: Colony forming unit

CI: Confidence interval

EGCG: (2)-epigallocatechin-3-O-gallate

EPA: Eicosapentaenoic acid

ERK: Extracellular signal-related kinases

γ-GCL: γ-glutamate cysteine ligase

GCLC: Glutamate cysteine ligase catalytic subunit

GRAS: Generally regarded as safe

GSH: Glutathione

GST: Glutathione S-transferase

GTC: Green tea catechin

HEMn: Primary human epidermal melanocytes

HETE: 12-hydroxyeicosatetraenoic acid

HMG-CoA: 3-hydroxy-3-methyl-glutaryl-coenzyme A

IC30: Concentration for 30% inhibition of substrate

IFN-γ: Interferon gamma

IgA: Immunoglobulin A

IgE: Immunoglobulin E

IgM: Immunoglobulin M

JNK: Jun nuclear kinase

LCC: A 10 mg free lutein stablized with 10% carnosic acid soft gel capsule

LGG: *Lactobacillus rhammosus* Goldin and Gorbach

MAPK: Mitogen-activated protein kinases

MED: Minimal erythema dose

MHC+ CD 1 1b+ cell types

NHEK: Normal primary human epidermal keratinocytes

NF-κβ: Nuclear factor kappa B

NMSC: Nonmelanoma skin cancer

NQO1: Nicotinamide adenine dinucleotide phosphate (NADPH) quinone oxidoreductase 1

Nrf2-ARE: Nuclear factor E2-related factor 2 (Nrf2)-antioxidant responsive element (ARE)

8-OHdG: 8-hydroxy-2′–deoxyguanosine

OR: Odds ratio

p38; p53: Tumor protein p38; p53

PGE2: Prostaglandin E2

RCT: Randomized controlled trial

ROS: Reactive oxygen species

SCC: Squamous cell carcinomas

SCORAD: Scoring Atopic Dermatitis

TEWL: Trans-epidermal water loss

Th1: Type 1 helper T lymphocyte

Th2: Type 2 helper T lymphocyte

TNC: A 5 mg lycopene with tomato phytonutrient complex including tocopherols and phytosterols soft gel capsule

TNF-α: Tumor necrosis factor α

USA: United States of America

UVA: ultraviolet A radiation

UVA1: Longwave UV radiation

UVB: ultraviolet B radiation

UVC: ultraviolet C radiation

UVR: ultraviolet radiation
